# Whole exome sequencing identified a novel *DAG1* mutation in a patient with rare, mild and late age of onset muscular dystrophy‐dystroglycanopathy

**DOI:** 10.1111/jcmm.13979

**Published:** 2018-11-18

**Authors:** Yi Dai, Shengran Liang, Xue Dong, Yanhuan Zhao, Haitao Ren, Yuzhou Guan, Haifang Yin, Chen Li, Lin Chen, Liying Cui, Santasree Banerjee

**Affiliations:** ^1^ Department of Neurology Peking Union Medical College Hospital Chinese Academy of Medical Sciences Beijing China; ^2^ School of Life Science and Biopharmaceuticals Guangdong Pharmaceutical University Guangzhou China; ^3^ Department of Cell Biology Tianjin Medical University Tianjin China; ^4^ Department of Cell Biology and Medical Genetics School of Medicine Zhejiang University Hangzhou China; ^5^ Neurosciences Center Chinese Academy of Medical Sciences Beijing China

**Keywords:** α‐dystroglycan, *DAG1* gene, MDDGC9, muscular dystrophy‐dystroglycanopathy, whole exome sequencing

## Abstract

Muscular dystrophy‐dystroglycanopathy (limb‐girdle), type C, 9 (MDDGC9) is the rarest type of autosomal recessive muscular dystrophies. MDDGC9 is manifested with an early onset in childhood. Patients with MDDGC9 usually identified with defective glycosylation of DAG1, hence it is known as “dystroglycanopathies”. Here, we report a Chinese pedigree presented with mild MDDGC9. The proband is a 64 years old Chinese man. In this family, both the proband and proband's younger brother have been suffering from mild and late onset MDDGC9. Muscle biopsy showed that the left deltoid muscle with an advanced stage of dystrophic change. Immunohistochemistry staining of dystrophin, α‐sarcoglycan, β‐sarcoglycan and dysferlin are normal. Molecular genetic analysis of the proband has been done with whole exome sequencing. A homozygous novel missense mutation (c.2326C>T; p.R776C) in the exon 3 of the *DAG1* gene has been identified in the proband. Sanger sequencing revealed that this missense mutation is co‐segregated well among the affected and unaffected (carrier) family members. This mutation is not detected in 200 normal healthy control individuals. This novel homozygous missense mutation (c.2326C>T) causes substitution of arginine by cystine at the position of 776 (p.R776C) which is evolutionarily highly conserved. Immunoblotting studies revealed that a significant reduction of α‐dystroglycan expression in the muscle tissue. The novelty of our study is that it is a first report of *DAG1* associated muscular dystrophy‐dystroglycanopathy (limb‐girdle), type C, 9 (MDDGC9) with mild and late age of onset. In Chinese population this is the first report of *DAG1* associated MDDGC9.

## 1 INTRODUCTION

Muscular dystrophies are a group inherited disorder characterised by gradual and progressive weakness of muscles.^1^ It shows an extreme genotypic and phenotypic heterogeneity.^2^ The normal function of human skeletal muscle needs both the intracellular sarcomeric proteins and extracellular matrix (ECM). Intracellular cytoskeleton and ECM are mechanically strongly linked together by a multimeric protein complex named as dystrophin–glycoprotein complex (DGC).^3^, ^4^ DGC is composed by intracellular, extracellular and transmembrane proteins. Hence, mutations in any of the proteins of the DGC complex exert recessive negative effects and finally leads to different forms of hereditary muscular dystrophies.^5^, ^6^

The dystroglycan (*DAG1*) gene is located in chromosome 3, comprises of six exons. *DAG1* gene encodes dystroglycan protein which is the key component of DGC and plays a significant role in linking dystrophin with other proteins of ECM to form and give mechanical support to the intracellular cytoskeleton. *DAG1* gene is evolutionarily highly conserved and dystroglycan is primarily expressed as a precursor protein. During post‐translational modification, dystroglycan protein has been cleaved at Ser654 by proteolysis to form α‐dystroglycan and β‐dystroglycan.^7^ In the ECM, the extracellular protein α‐dystroglycan is linked with laminin α2. The membrane glycoprotein β‐dystroglycan is bind with dystrophin inside the cell and also linked with α‐dystroglycan extracellularly. Dystrophin is involved in interaction with actin cytoskeleton. Dystroglycans are also involved in signalling pathways by interacting with signalling proteins.^8^, ^9^

During post‐translational modification, dystroglycan is glycosylated for normal functioning, otherwise it will lose specific interaction with ligands.^4^ Till now, mutations of eight human genes are associated with abnormal or defective glycosylation of dystroglycan.^10^, ^11^ Recently, they have been collectively designated as “muscular dystrophy‐dystroglycanopathies(MDDG)”. In addition, MDDG are divided into three types: type A (the most severe, WWS and MEB included); type B (intermediate, congenital muscular dystrophy without brain and eye anomalies included); type C (the mildest, limb‐girdle muscular dystrophy included). Besides the deficiency in glycosylation of dystroglycans, a significant reduction in dystroglycan protein level is also evident in several patients with “dystroglycanopathies”.^12^ Dystroglycan interacts with several intracellular and extracellular proteins or signalling molecules. Intracellularly, β‐dystroglycan interacts with dystrophin, utrophin, DRP2 and other signalling molecules by its C‐terminal domain.^13^ In contrast, extracellularly, α‐dystroglycan interacts with laminin globular (LG) domains of laminin α2, neurexin, perlecan and agrin by glycosyl residue.^14^, ^15^

In our present study, we describe a three generation Chinese family with MDDGC9. Comprehensive clinical evaluation has been done, including muscle biopsy and pathological study. Whole exome sequencing identified a homozygous missense mutation in *DAG1* gene in the proband. Immunoblotting study identified that significantly decreased expression of α‐dystroglycan in the proband's muscle. *DAG1* related muscular dystrophy is very rare and till now only 10 mutations have been reported in *DAG1* gene to be associated with muscular dystrophy. So, our present study not only expand the mutational spectrum of the *DAG1* gene, but also emphasise the significance of whole exome sequencing for rapid, accurate and cost‐effective approach for identifying the novel mutation of candidate genes associated with genetically and phenotypically extremely heterogeneous hereditary muscular dystrophy. This is the first report of *DAG1* associated MDDGC9 in China as well as the first report of late age of onset of MDDGC9 worldwide.

## 2 METHODS

### 2.1 Patients

**Figure 1:**
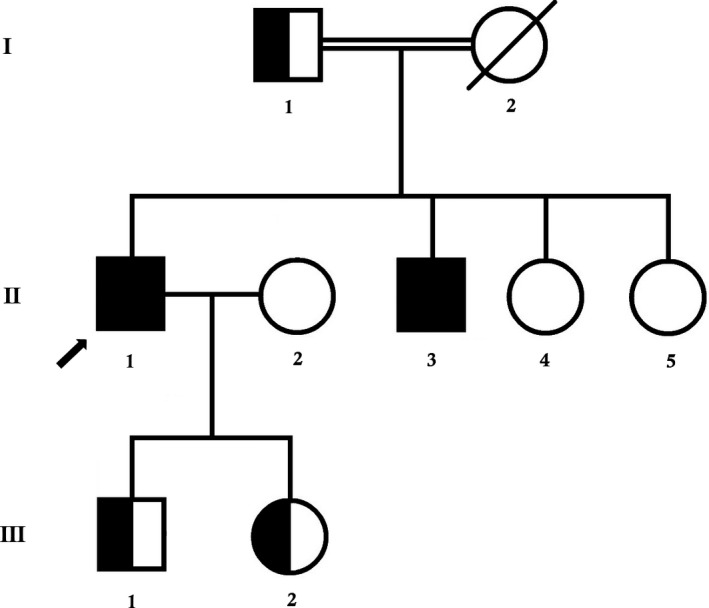
Pedigree of the family. The filled symbol indicates the patient (proband), and the half‐filled symbols show the carrier parents, who were heterozygous carriers but were unaffected. The arrow points to the proband

In this study, a three generation Han Chinese family (Hubei province) with mild autosomal recessive MDDGC9 was enrolled in the Department of Neurology, Peking Union Medical College Hospital, Beijing, China (Figure 1). The study was approved by the ethics committee of the Peking Union Medical College Hospital, Beijing, China, in accordance with the recommendations of the Declaration of Helsinki. Written informed consent has been obtained from all the participant of this study.

### 2.2 Clinical evaluation

We performed comprehensive clinical examinations related to neuromuscular disorders. We explored routine blood tests including serum creatine kinase level. Electrophysiological study and muscle biopsy were performed. The muscle pathology procedures included standard staining and the immunohistochemistry staining of dystrophin‐N, dystrophin‐R, dystrophin‐C, α‐sarcoglycan, β‐sarcoglycan and dysferlin‐C (mouse monoclonal antibody, Novocastra, US).

### 2.3 Whole exome sequencing

**Figure 2:**
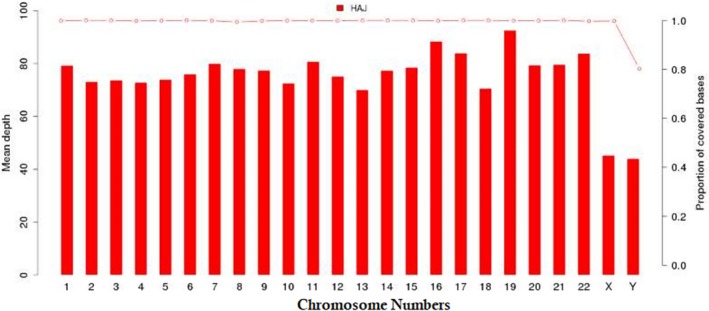
Coverage depth (left coordinates) and coverage (right coordinates) for each chromosome

The genomic DNA was extracted from peripheral blood, randomly fragmented and sheared into fragments of 180‐280 bp in length using a Covaris crusher. After the ends were repaired and A tails were added, DNA fragments were ligated to the ends of the fragments to prepare DNA libraries. The exome was enriched using Agilent's SureSelect Human AII Exon V5 Kit, with up to 543872 biotins after library pooling with a specific index. The labelled probes were hybridised in liquid phase. Then 334378 exons of 20965 genes were captured using streptomycin‐containing magnetic beads. The libraries were linearly amplified by PCR and subjected to library quality tests. After passing the test, they were carried out high‐throughput deep sequencing on the Illumina HighSeq 2500 (Figure 2).

Burrows‐Wheeler Aligner software (version 0.59) was used to align the sequencing reads with the GRCh37.p10. Then the aligned reads were realigned and recalibrated by GATK Indel Realigner and the GATK Base Recalibrator respectively (broadinstitute.org/). GATK UnifiedGenotyper (broadinstitute.org/) was used for identifying the single‐nucleotide variants (SNV) and small insertions or deletions (indel). Finally, Consensus Coding Sequences Database (20130630) was used for variant annotation. According to the minor allele frequency with a cut‐off value of <0.05 in four databases (dbSNP, HapMap, 1000 Genome Project and in‐house Chinese local database), we filtered and selected the variants. Classification of variants (pathogenic, likely pathogenic, VUS and likely benign and benign) has been done according to the variant interpretation guidelines of American College of Medical Genetics and Genomics (ACMG).^16^ Finally, we compared the variants found in patient and other affected and unaffected (carrier or non‐carrier) family members. Gene function has been established from the previously published articles and OMIM database.

**Figure 3:**
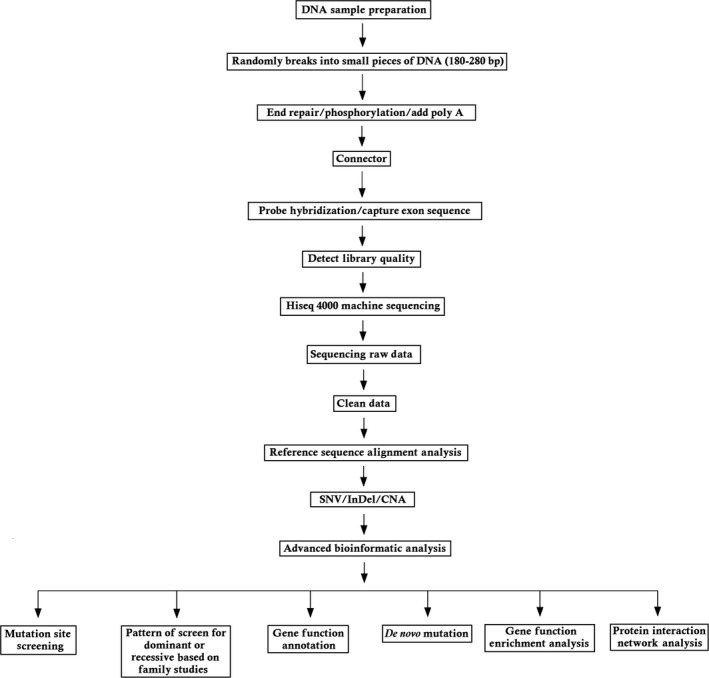
The detailed variant interpretation pipeline

The detailed variant interpretation process is described in Figure 3.

### 2.4 Sanger verification and co‐segregation analysis

Sanger sequencing has been performed for validating the variants identified by whole exome sequencing. The primers are as follows; F‐ 5′‐GCTGGCCATACGGACTCATCC‐3′, R‐ 5′‐GCCTAGCTTACTATAGGGCCG‐3′. The reference sequence NM_001177639 of *DAG1* was used.

### 2.5 Immunoblotting of α‐dystroglycan

Muscle sections were prepared and lysed by using lysis buffer and separated by 10% SDS‐PAGE followed by transfer of these gels to PVDF membrane. Skimmed milk (5%) was used for blocking the membrane and mouse monoclonal antibody was used to probe α‐dystroglycan. α‐actinin was used as a loading control. Goat antimouse IgG was used to detect the bound primary antibody and performed western blot.^17^, ^18^

### 2.6 Data availability

All data used for the analyses in this report are available through Genome Sequence Archive, BIG Data Center in Beijing Institute of Genomics (GEO) [URL: http://bigd.big.ac.cn/gsub/submit/gsa/list]. Accession number: CRA000997.

## 3 RESULTS

### 3.1 Clinical findings

The proband is a 64 years old man. The proband was born from a consanguineous Chinese family (Figure 1). The onset symptom was slight pelvic girdle muscle weakness when he was about 22 years old. Three years later, he went to the local hospital and was diagnosed as muscular dystrophy. Without specific therapy, the disease progressed steadily and slowly. Although his athletic performance is limited, he had an almost normal life. When he was referred to our hospital, he still can walk by himself. On the neurological physical examination, there is no facial or tongue weakness. The muscle of the limbs is atrophied, especially in proximal part. Muscle strength in Medical Research Council (MRC) is proximal 3/5, distal 4/5 in lower limbs and proximal 3+/5, distal 4/5 in upper limbs. He was unable to stand from a lying position without help. He has a younger brother (II‐2) who had similar symptoms.

The results of blood tests indicated serum muscle enzymes were slightly elevated (CK 1049‐1184U/L (24‐195), LDH 323 U/L↑(0‐250), AST 42U/L↑(15‐40), ALT 30U/L (9‐50)), while complete blood counts (WBC 7.52x10^9^, Neut% 59.6%, Ly% 31.8%, RBC 4.84 × 10^12^, Hb 150 g/L, PLT 287 × 10^9^) and coagulation testing were normal. HBsAg, HIV‐Ab, HCV‐Ab and TP‐Ab were negative.

Nerve conduction velocity and electromyogram tests showed myogenic changes with normal peripheral nerve conduction.

### 3.2 Pathological result of muscle biopsy

**Figure 4:**
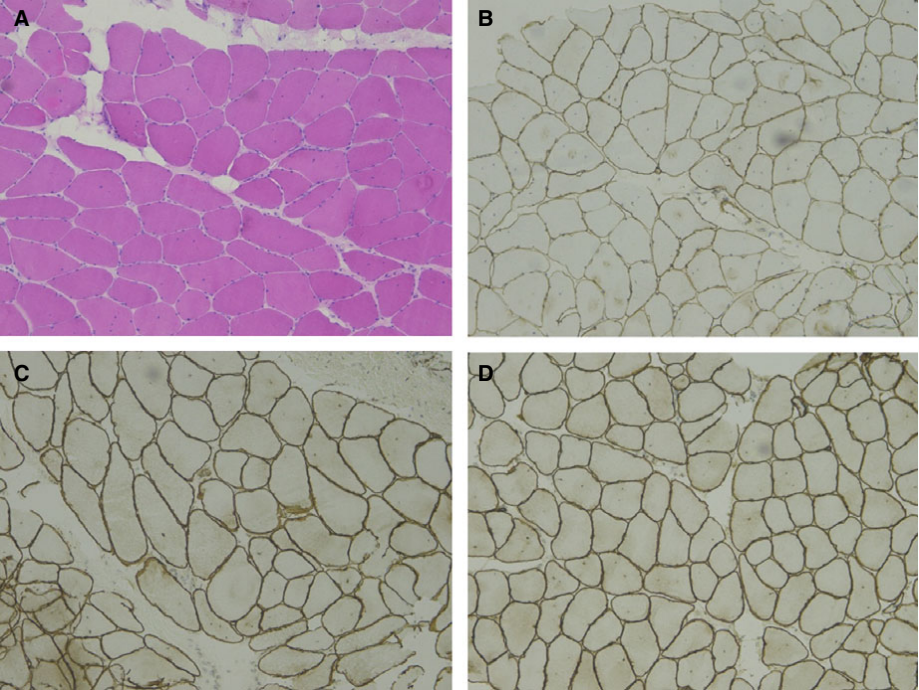
Muscle Biopsy. A, haematoxylin‐eosin staining; B, IHC staining of dystrophin‐N; C, IHC staining of α‐sarcoglycan; D, IHC staining of β‐sarcoglycan

The pathological findings of left deltoid muscle illustrated advanced stage of dystrophic change. There is a predominance of type I fiber. Immunohistochemistry staining of dystrophin, α‐sarcoglycan, β‐sarcoglycan and dysferlin are normal (Figure 4).

### 3.3 Identification of a novel mutation in *DAG1*

**Figure 5:**
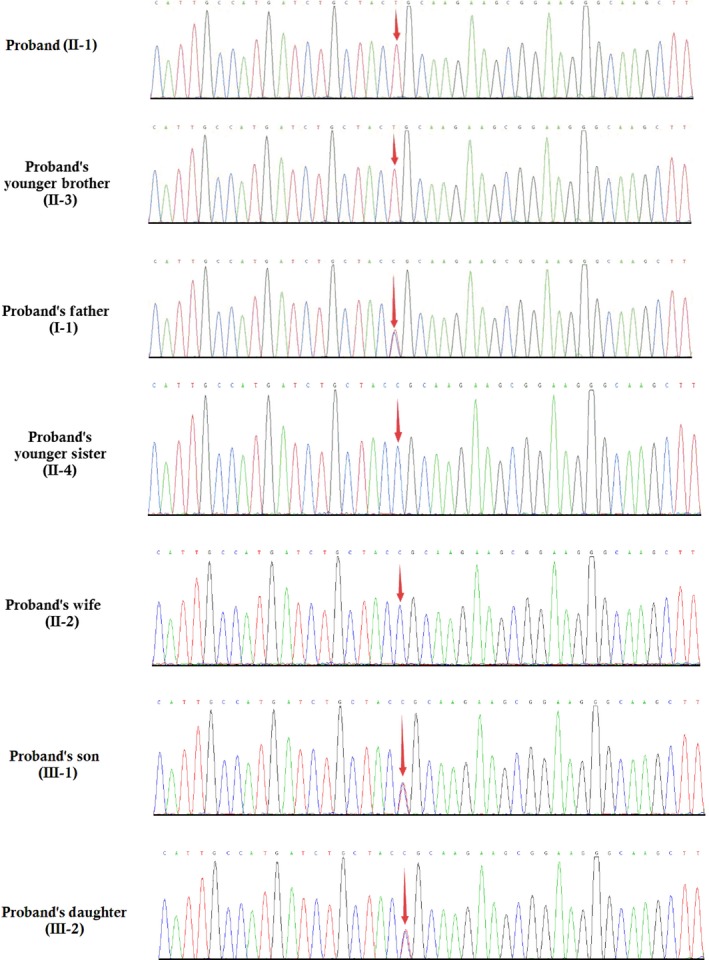
Partial DNA sequences in the *DAG1* gene by Sanger sequencing of the family

A novel homozygous missense mutation (c.2326C>T) in exon 3 of the *DAG1* gene has been identified in the proband (II‐1) and proband's younger brother (II‐3) by whole exome sequencing (Figure 5). This missense mutation leads to replacement of arginine by cysteine (p.Arg776Cys) at the position of 776 in dystroglycan protein. Sanger sequencing confirmed that proband's father (I‐1), proband's son (III‐1) and proband's daughter (III‐2) were carrying this mutation in a heterozygous form. Proband's wife (II‐2) and two younger sisters (II‐4 and II‐5) were devoid of this mutation.

This mutation is co‐segregated well among the affected patients and unaffected carrier in this family but absent in unaffected family members and 200 healthy control. We have not identified this mutation in Human Gene Mutation database (HGMD, www.hgmd.cf.ac.uk/) and MIM (https://www.omim.org). The heterozygous prevalence of this missense mutation is very low, 0.000025 in ExAC (http://exac.broadinstitute.org) and 0.000022 in gnomAD (http://gnomad.broadinstitute.org) respectively. There is no homozygous prevalence of this missense mutation reported in both databases.

**Figure 6:**
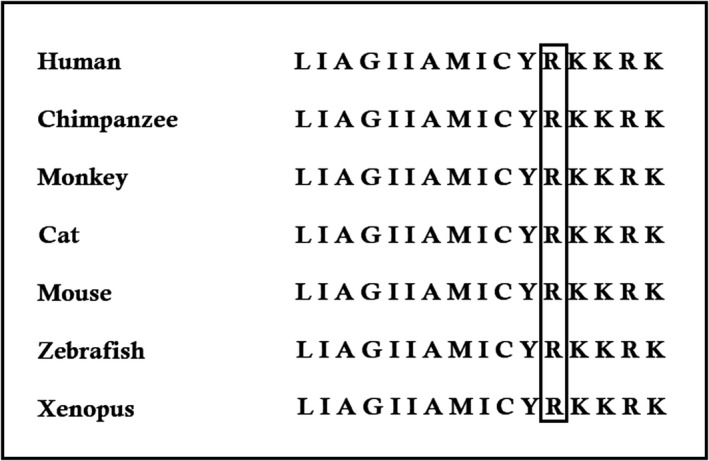
Multiple amino acid sequence alignment of the wild‐type protein encoded by WDR62 (Homo sapiens) (GenBank Accession: NM_001177639.2) with Chimpanzee (Pan troglodytes) (GenBank Accession: XM_001164638.4), rhesus monkey (Macaca mulatta) (GenBank Accession: NM_001261351.1), Cat (Felis catus) (GenBank Accession: NM_001130839.1), Mouse (Mus musculus) (GenBank Accession: NM_001276481.1), Zebrafish (Danio rerio) (GenBank Accession: NM_173274.1), and Clawed frog (Xenopus laevis) (GenBank Accession: NM_001177643). The black box showed that amino acids 776 are conserved across these species

In silico analysis was performed to understand and predict the significance of this mutation. In dystroglycan protein, the p.Arg776 is evolutionarily highly conserved among different species (Figure 6). Hence, we can predict that mutation in this residue can exert a dominant negative effect to the dystroglycan protein structure which renders the normal function of the dystroglycan protein and leads to the disease phenotype.

Online mutation prediction softwares, Mutation Taster2, PolyPhen‐2 and I‐Mutant 2.0 predicted this mutation as “disease causing,” “probably damaging” and “decreasing stability of the DAG1 protein” respectively.^19^, ^20^, ^21^

### 3.4 Immunoblotting of α‐dystroglycan

**Figure 7:**
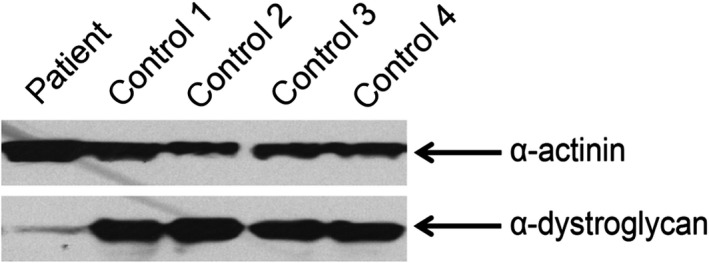
The controls are four C57 mice. The Western blotting analysis of the muscle tissue from the patient and the controls shows the α‐dystroglycan is significantly reduced in the proband

The α‐dystroglycan in the muscle from the patient was identified with significantly reduced expression compared to other control muscle samples (Figure 7). This finding implies the homozygous missense mutation in *DAG1* gene impairs the dystroglycan protein structure and function.

## 4 DISCUSSION

Among all the types of inherited muscular dystrophies, MDDGC9 is the rarest form. However, in patients with MDDGC9, defective or abnormal glycosylation or hypoglycosylation of α‐dystroglycan leads to the loss of extracellular interaction between α‐dystroglycan with laminin which finally results into progressive weakness of skeletal muscle.^5^ Here, we identified a three generation Han Chinese family with mild and late onset MDDGC9. Whole exome sequencing and Sanger sequencing identified a novel homozygous *DAG1* mutation which is co‐segregated well among patients and the unaffected carriers in this family. Functional characterisation identified that this missense mutation causes significant reduction in expression of α‐dystroglycan compared with control. Hence, we again establish that the important role of α‐dystroglycan in normal functioning of the muscle and mutation of it leads to muscular dystrophy.

The novel missense mutation in *DAG1* gene; a single nucleotide transition c.2326C>T in exon 3 which leads to the replacement of arginine by cysteine at the position of 776 (p.Arg776Cys). Replacement of basic, polar and positive charged arginine by sulphur containing, non‐polar and neutral cysteine at the evolutionarily highly conserved position (p.Arg776) possibly exerts a recessive negative effect which finally results into muscular dystrophy. The positive charge of arginine forms favourable electrostatic interactions with the negative phospholipid head‐group. Hence, arginine mutated to cysteine will perturb the normal electrostatics. In addition, R776 is the first cytoplasmic residue of β‐dystroglycan. R776 also forms a part of the β‐dystroglycan nuclear localisation signal.^22^ The RKKRK motif is also a docking site for MAPK.

In addition, both genotypically and phenotypically, dystroglycanopathy is extremely heterogeneous. The clinical diagnosis of these three generation Chinese family with Limb‐girdle muscular dystrophy has been done based on the classification system 22 Jimenez‐Mallebrera et al, reported that clinical severity of MDDGC9 is not directly correlated with the glycosylation level of α‐dystroglycan.^23^ Till date, only 18 genes have been identified to be associated with dystroglycanopathies. 24, 25, 26, 27 Among these 18 genes, germline mutation of *DAG1* mutation is associated with primary dystroglycanopathy in limb‐girdle muscular dystrophy and muscle‐eye‐brain disease.^10^, ^11^

In addition, due to extreme genotypic and phenotypic heterogeneity, clinical diagnosis through genetic screening for the patients with dystroglycanopathy, is really a big challenge. In this study, we performed whole exome sequencing (WES) for identifying the candidate gene with novel mutation. Our present study strongly emphasises the significance of WES for rapid, accurate and cost‐effective approach for identifying the causative gene with pathogenic mutation.

However, *DAG1* gene associated MDDGC9 is a very rare dystroglycanopathy and till now less than 10 cases reported worldwide. Here, the clinical symptoms of patients are also very different from those previous reports as the clinical phenotype of our studied patient is very mild and very slowly progressing with late age of onset. To be the best of our knowledge, this is the first description of a potentially pathogenic mutation of the *DAG1* gene associated with MDDGC9 in China.

## CONFLICT OF INTEREST

The authors confirm that there are no conflicts of interest.

## AUTHOR CONTRIBUTIONS

Y.D., S.L. conducted acquisition, analysis and interpretation of data, and drafted and edited the manuscript. S.B., L.Cu., L.C., Y.D. and S.L. supervised all aspects of this study including study design, data interpretation, and drafted and edited the manuscript. X.D., Y.Z. and H.R. made WES pipeline and analysed the data. Y.D., Y.G. and H.Y. selected patients and performed WES. Y.D. and S.L. collected clinical information of the patient. S.B., L.Cu., L.C., C.L. and Y.D. supervised manuscript preparation and edited the manuscript.
